# Recycled Automotive and Construction Wastes in Three-Layer Particleboards: Thermophysical Properties, Sound Absorption, and Radiant-Heat Mass-Loss Behavior

**DOI:** 10.3390/polym18161997

**Published:** 2026-08-17

**Authors:** Rupali Tiwari, Anna Darabošová, Iveta Čabalová, Miroslav Němec, Martin Zachar, Tereza Jurczyková, Lubos Kristak

**Affiliations:** 1 Faculty of Wood Sciences and Technology, Technical University in Zvolen, T. G. Masaryka 24, 960 01 Zvolen, Slovakia; tiwari@tuzvo.sk (R.T.); xdarabosova@is.tuzvo.sk (A.D.); nemec@tuzvo.sk (M.N.); zachar@is.tuzvo.sk (M.Z.); kristak@tuzvo.sk (L.K.); 2 Institute of Physics, Slovak Academy of Sciences, Dúbravská cesta 9, 845 11 Bratislava, Slovakia; 3 Faculty of Forestry and Wood Sciences, Czech University of Life Sciences Prague, Kamýcká 1176, 165 21 Prague, Czech Republic; jurczykova@fld.czu.cz; 4 Academy of Fine Arts in Prague, Painted Artworks and Polychrome Statues Restoration, U Akademie 4, 170 00 Prague, Czech Republic

**Keywords:** particleboard, recycled automotive plastics, waste rubber, electrical-cable waste, transient plane source, thermal diffusivity, sound absorption, radiant heat, polarizing optical microscopy

## Abstract

Recycled polymer-rich residues can alter several functions of wood-based panels, but claims of multifunctionality require property-specific evidence and transparent treatment of replication. Three-layer spruce particleboards were therefore prepared with 10 wt.% painted or unpainted polypropylene bumper granules, high-density polyethylene fuel-tank granules, tire rubber, seal-and-carpet residues, or electrical-cable fractions in the core layer. Two hybrid formulations contained 10 wt.% recycled rubber-rich filler plus 10 wt.% expandable graphite. Transient plane source measurements were evaluated at the specimen-pair level (two pairs per formulation); normal-incidence sound absorption and radiant-heat mass loss were complementary descriptive screens because the archived datasets contained one spectrum or one exposed specimen per formulation. Mean thermal conductivity varied only from 0.1908 to 0.2075 W m^−1^ K^−1^ (−5.3% to +3.0% relative to the reference). The graphite hybrids showed the clearest change in transient response: thermal diffusivity increased by 19.6–20.1%, whereas volumetric heat capacity decreased by 14.5–16.7% and thermal effusivity by 6.5–8.7%. SC10G10 had the highest mean absorption coefficient over 126–6400 Hz (0.210; +46.2%) and the lowest mass loss after 600 s at 30 kW m^−2^ (37.48%; −17.72%). Macroscopic and polarizing optical images showed formulation-dependent filler distribution and visible interfacial spaces, but they did not establish bonding mechanisms or quantify porosity. The results identify promising formulation-dependent responses while also defining the replication and structural measurements needed before application-level claims can be made.

## 1. Introduction

The building and construction sector remains a major consumer of energy and a substantial source of greenhouse gas emissions. Recent global assessments indicate that the sector accounts for approximately 32% of global energy use and 34% of global carbon dioxide emissions [1]. A considerable share of building energy demand is associated with maintaining indoor thermal conditions; therefore, the heat-transfer characteristics of building envelopes and interior materials directly affect operational energy consumption and indoor comfort [2,3]. In addition to thermal conductivity, transient properties, such as thermal diffusivity, volumetric heat capacity, and thermal effusivity, are important because building materials experience changing thermal conditions rather than an exclusively steady-state heat flow. The transient plane source method can determine thermal conductivity and diffusivity from one transient response, and is well-suited to comparative measurements on heterogeneous composites [4,5].

At the same time, end-of-life vehicles are a source of heterogeneous polymeric waste, including polypropylene bumpers, polyethylene fuel tanks, tires, seals, carpets, and insulated cables. More than six million vehicles reach the end of their service life annually in Europe [6]. Directive 2000/53/EC established minimum targets of 85% for reuse and recycling and 95% for reuse and recovery by average vehicle weight [7]. Although metallic fractions are recovered efficiently, the closed-loop or high-value recycling of mixed polymer fractions remains difficult. Incorporation into wood-based panels is one possible material-recovery route, provided that the resulting performance and limitations are reported without treating all waste streams as equivalent.

Particleboard is widely used in furniture and interior construction because small particles and secondary lignocellulosic raw materials can be consolidated using established pressing technology [8,9]. Recycled wood, agricultural residues, and post-consumer polymers have all been incorporated into particleboards [8,10,11]. Their effects depend on filler chemistry, particle size and shape, dispersion, interfacial contact, adhesive distribution, density, and pore structure. A filler can consequently improve one response while leaving another unchanged or impairing load transfer.

Most studies of waste-modified particleboards emphasize density, water absorption, thickness swelling, bending, and internal bond strength [8,10,11]. Thermal conductivity is sometimes reported as a single steady-state property, although conductivity alone does not describe the rate of temperature equalization or heat storage. Work combining transient thermophysical measurements with normal-incidence sound absorption, controlled radiant-heat mass loss, and direct structural observations remains comparatively limited [12,13].

Members of the present research group have previously studied particleboards containing tire rubber or seal-and-carpet mixtures, with an emphasis on ignition-related and calorimetric responses [13]. A separate study examined three-layer boards containing recycled painted and unpainted polypropylene bumpers or high-density polyethylene fuel tanks, focusing on mechanical and physical properties, dimensional stability, and volatile organic compound emissions [14]. Čabalová et al. examined single-layer rubber- and tire-modified boards produced in a separate experimental program, including their thermophysical and acoustic properties [15]. More recently, Szul and Darabošová conducted a preliminary acoustic screening using a separate, independently manufactured series of three-layer panels [16]. Their proof-of-concept study indicated that selected recycled rubber and polymer fractions warranted broader functional investigation. The panels, test specimens, acoustic records, and numerical results reported in this preliminary study are not reused here. All cited earlier studies examined panels and test specimens prepared independently of the new panel series investigated in the present work.

The present study therefore reports an independent follow-on experiment, and compares ten formulations from a newly manufactured panel series within one manufacturing system. Its first objective was to determine whether replacing core wood particles with individual recycled automotive or construction waste fractions changes thermal conductivity, diffusivity, volumetric heat capacity, effusivity, and specific heat capacity. The second was to relate these measurements descriptively to sound absorption, radiant-heat mass-loss behavior, and mesoscale structure. SC10G10 and T10G10 were treated as a distinct hybrid group because each contained 10 wt.% recycled rubber-rich filler and 10 wt.% expandable graphite; their responses cannot be attributed to the waste fraction alone.

## 2. Materials and Methods

### 2.1. Raw Materials and Waste Preparation

Industrial spruce (*Picea abies* L.) particles were supplied by Kronospan s.r.o. (Zvolen, Slovakia). Core particles were 0.25–4.0 mm and surface particles 0.25–1.0 mm. Before manufacture, their moisture content was adjusted to 4.0 ± 0.5% and determined according to EN 322 [17]. Finished-board equilibrium moisture content at the time of functional testing was not measured separately.

Painted and unpainted polypropylene bumper parts and high-density polyethylene fuel tanks were obtained from ALUEX s.r.o. (Zvolen, Slovakia). Tire rubber, mixed seals and carpets, and electrical-cable fractions were supplied by AVE Kechnec s.r.o. (Košice, Slovakia). Polymer- and rubber-containing residues were cut into smaller pieces, cleaned, and granulated at the Technical University in Zvolen with a Profing DP 11–240/350 shredder (Profing, Piestany, Slovakia) and Holzmann ABS 1080 dust extractor (Holzmann, Asslach, Austria). Granules retained between 1 and 4 mm were selected using a Retsch AS 200 digit cA analytical sieve (Retsch, Haan, Germany). The archived production record did not specify a liquid cleaning medium, cleaning time, or post-cleaning drying schedule; the earlier, less specific word “washed” has therefore not been retained.

Kronores CB 1100 F urea–formaldehyde resin (Kronospan s.r.o., Zvolen, Slovakia; 67.1% solids, viscosity 460 mPa s, gel time 55 s, pH 8.6) was used as binder. A 47 wt.% ammonium nitrate solution was added at 1 wt.% relative to resin solids, and a 30 wt.% paraffin emulsion was added at 1 wt.% relative to oven-dry furnish. Expandable graphite was used in the two hybrid formulations.

### 2.2. Particleboard Manufacture

**Figure 1:**
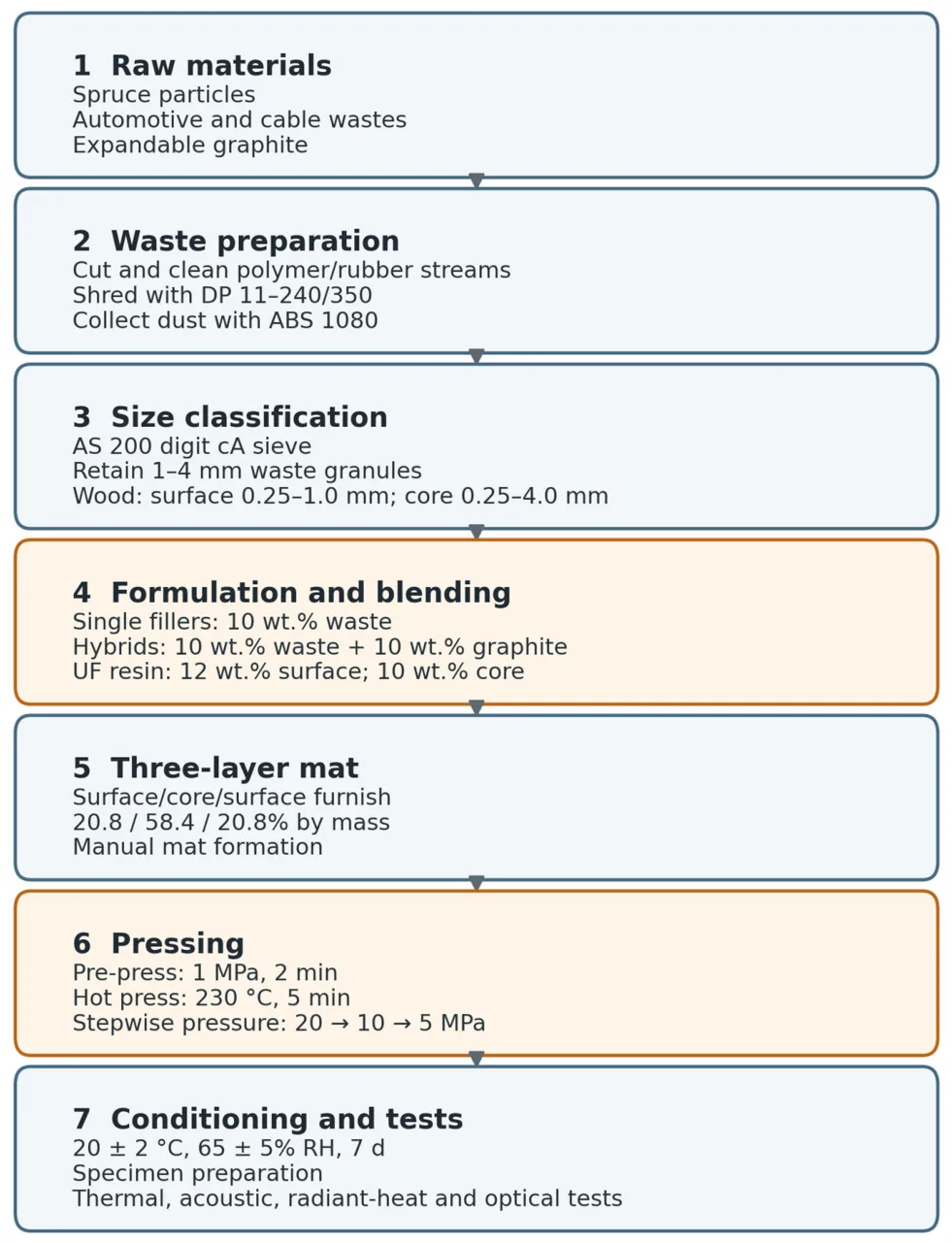
Manufacturing and testing workflow. Percentages refer to oven-dry core solids unless otherwise stated.

Manufacturing and testing workflow is in Figure 1. For the single-filler formulations, recycled filler replaced 10 wt.% of the oven-dry core furnish. In the hybrids, 10 wt.% tire rubber or seal-and-carpet residue and 10 wt.% expandable graphite were included, leaving 80 wt.% spruce particles in the dry core solids. The total core furnish was held constant, and no recycled filler was added to the surface layers. UF resin was applied at 10 wt.% to the core and 12 wt.% to the surface furnish, as based on oven-dry particles.

The blended furnish was formed manually into three-layer mats. The archived layer masses correspond to a surface/core/surface proportion of 396:1110:396, or 20.8:58.4:20.8% by mass. Mats were pre-pressed at 1 MPa for 2 min and then hot-pressed in a CBJ 100–11 laboratory press (TOS Rakovník, Rakovník, Czech Republic) at 230 °C for 5 min using a stepwise 20 → 10 → 5 MPa pressure profile. Three panels were produced for each formulation. Nominal finished dimensions were 600 × 400 × 18 mm. Panels were conditioned for 7 d at 20 ± 2 °C and 65 ± 5% relative humidity before specimen preparation. These panels constituted a new production series prepared specifically for the present experiment; no panels or test specimens from the related studies [13,14,15,16] were reused.

### 2.3. Formulations and Density

Codes and core compositions are given in Table 1. Density was determined according to EN 323 [18] on the four 100 × 100 mm specimens associated with each thermophysical series. Accordingly, Table 1 reports mean ± sample standard deviation for n = 4 specimens rather than one selected specimen value. Cable labels reproduce the source categories and do not constitute regulatory fire classifications.

### 2.4. Thermophysical Characterization and Data Reduction

Thermophysical properties were measured by the transient plane source (TPS) method, in accordance with ISO 22007-2:2022 [19] and the established TPS framework [20], using a Hot Disk TPS 2500 S analyzer (Hot Disk AB, Gothenburg, Sweden) in isotropic mode. The Kapton-insulated nickel double-spiral sensor (Hot Disk AB, Gothenburg, Sweden) had a radius of 6.48 mm and acted simultaneously as a planar heat source and resistance thermometer.

Four 100 × 100 × 18 mm specimens were prepared per formulation and combined into two specimen pairs. For each transient, the sensor was centered between the plane faces of two specimens of the same formulation. The heating power was 50 mW, and the acquisition time was 320 s. The archived dataset contained four or five complete numerical TPS records for each pair. All complete records were retained, and their arithmetic mean was first calculated for each pair so that technical repeats did not inflate the experimental sample size.

For each formulation, the reported mean and sample SD were then calculated across the two pair means (n = 2 specimen pairs). Volumetric heat capacity (*C_v_*), thermal effusivity (e), and specific heat capacity (*c_p_*) were calculated at the pair level using Equations (1)–(3). Pair density was the arithmetic mean of the two constituent specimen densities. Because n = 2 is insufficient for a robust normality assessment or formulation-wise inferential comparison, the thermophysical analysis is descriptive.

*C_v_* = *λ*/*α* = *ρc_p_* (1)

*e* = √(*λC_v_*) (2)

*c_p_* = *C_v_*/*ρ* (3)

Here, *λ* is thermal conductivity (W m^−1^ K^−1^), α is thermal diffusivity (m^2^ s^−1^), *C_v_* is volumetric heat capacity (J m^−3^ K^−1^), *e* is thermal effusivity (W s^1^ᐟ^2^ m^−2^ K^−1^), *c_p_* is specific heat capacity (J kg^−1^ K^−1^), and *ρ* is density (kg m^−3^). The software-reported thermal probing depths in the archived records were approximately 11–15 mm and thus did not exceed the 18 mm thickness of either specimen in the sandwich. Surface roughness was not quantified, and the archived record did not verify the use of a contact medium; these contact-related factors remain sources of measurement uncertainty.

### 2.5. Normal-Incidence Sound Absorption

The normal-incidence sound absorption coefficient was measured using the transfer-function method according to ISO 10534-2:2023 [21]. A Brüel & Kjær Type 4206 impedance tube (Brüel & Kjær, Nærum, Denmark) was connected to a PULSE™ 14 acquisition system and Type 3050 LAN-XI module with two phase-matched microphones. A 100 mm tube covered the lower-frequency range and a 29 mm tube the higher-frequency range. Circular specimens were machined to the corresponding internal diameters and fitted to limit peripheral leakage.

The supplied spectra had a 2 Hz resolution. Analysis was restricted to the recorded 126–6400 Hz interval; 126 Hz was the first archived frequency at or above the nominal 125 Hz lower limit. Means were calculated for 126–1600 Hz, 1602–6400 Hz, and the complete 126–6400 Hz range. All formulations had the same nominal 18 mm thickness. The archive contained one combined spectrum per formulation and no replicate spectra; consequently, acoustic comparisons are descriptive and no acoustic error bars or significance tests are reported. The acoustic records analyzed here were obtained from specimens cut from this new panel series and are independent of the preliminary measurements reported by Szul and Darabošová [16].

### 2.6. Radiant-Heat Mass-Loss Test

A modified radiant-heat apparatus described in Slovak Utility Model No. 9373 was used [22]. It comprised a 1000 W ceramic infrared heater, a heat-load control unit, and a digital balance with ±0.01 g resolution. Specimens measuring 50 × 40 × 18 mm were exposed to 30 kW m^−2^ for 600 s, and the mass was recorded every 10 s. The supplied archive contained one complete mass-loss series per formulation; the results therefore represent a comparative screening test and not a fire-resistance rating or material classification.

Percentage mass loss, *δ*(*t*), and the interval mass-loss rate, *v*(*t_i_*), were calculated as follows:

*δ*(*t*) = [*m*_0_ − *m*(*t*)]/*m*_0_ × 100 (4)

*v*(*t_i_*) = [*δ*(*t_i_*) − *δ*(*t_i_*_−1_)]/Δ*t* (5)

where *m*_0_ is the initial specimen mass, *m*(t) is mass at exposure time *t*, and Δ*t* = 10 s. Peak and average interval mass-loss rates were derived from the recorded series. No uncertainty is reported because only one specimen series was available per formulation.

### 2.7. Macroscopic and Polarizing Optical Examination

Specimens were cut from the particleboards using a handheld band saw to expose cross-sectional and longitudinal surfaces. Sections approximately 5 mm thick were examined macroscopically to assess the three-layer structure, filler distribution, structural heterogeneity, and the occurrence of visible spaces within the core layer.

Polarizing optical microscopy was performed with a Nikon Eclipse Ni microscope (Nikon, Tokyo, Japan). Digital images were acquired at 40× magnification with a spatial resolution of 0.85 μm px^−1^ and processed with the Deep Focus module in QuickPHOTO 3.5 (Promicra, Prague, Czech Republic) to extend the effective depth of field. Observations addressed filler morphology, distribution, integrity, and visible filler–wood gaps. The images were interpreted qualitatively; they do not quantify porosity, interfacial adhesion, or chemical bonding.

## 3. Results

### 3.1. Thermophysical Properties

Table 2 summarizes the pair-level thermophysical results. The recalculation used every complete archived transient record, averaged technical repeats within each specimen pair, and then treated the two pair means as n = 2. It also corrected the CEN10/CIF10 label assignment present in the earlier aggregation. The uncertainty intervals therefore describe between-pair variation and are not instrument-only repeatability estimates.

**Figure 2:**
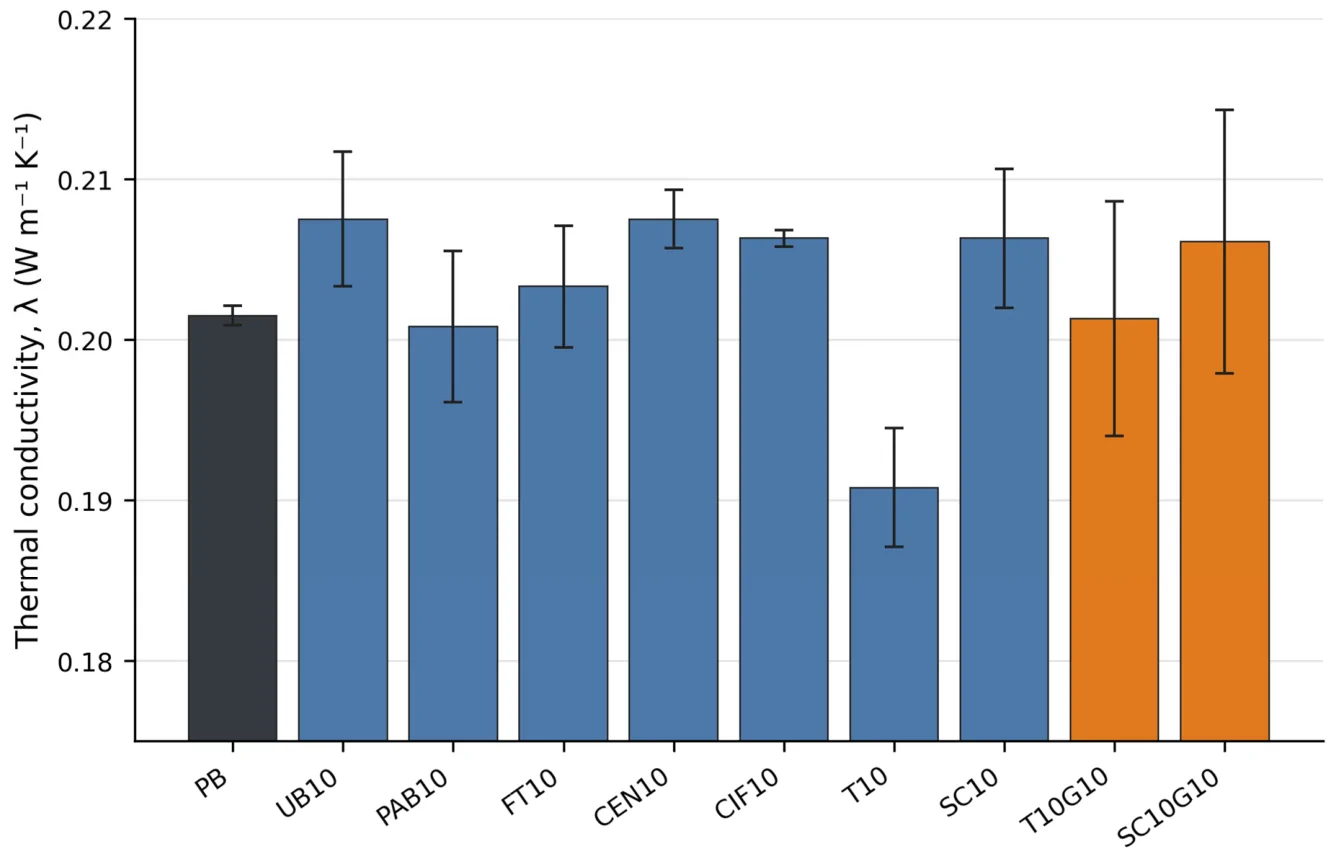
Thermal conductivity of the reference and waste-modified particleboards. Bars show formulation means; capped lines show sample SD across two specimen-pair means (n = 2).

Mean thermal conductivity occupied a narrow 0.1908–0.2075 W m^−1^ K^−1^ interval (Figure 2). Relative to PB, the largest decrease was 5.3% for T10; the largest increases were 3.0% for UB10 and CEN10. The absolute differences were small compared with the substantial between-specimen density dispersion in Table 1, and several pair-level SD intervals overlapped. The data therefore support a description of modest shifts and not a ranking of statistically distinct formulations.

**Figure 3:**
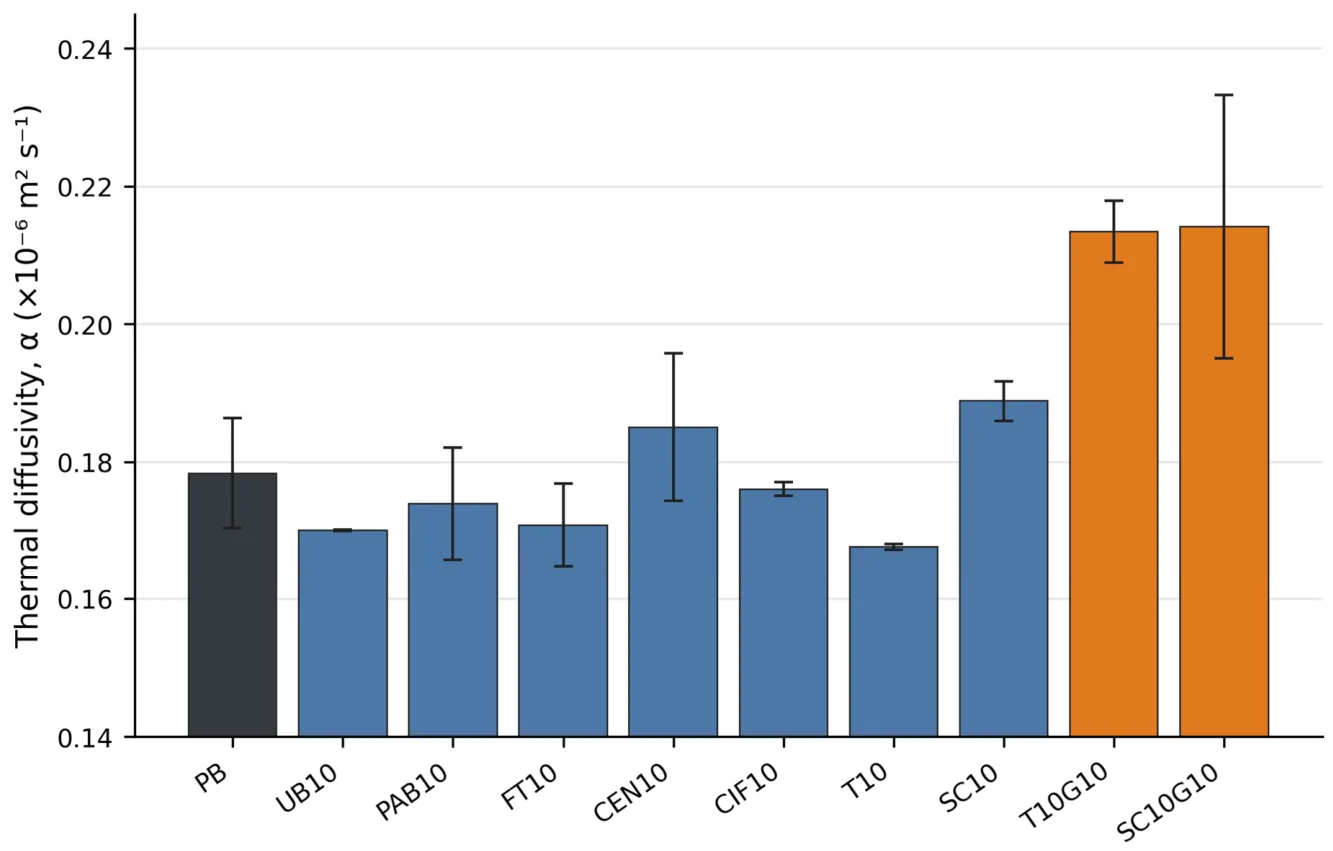
Thermal diffusivity. Bars and capped lines denote mean ± sample SD across two specimen-pair means (n = 2).

Thermal diffusivity varied more strongly (Figure 3). SC10G10 and T10G10 reached 0.2141 and 0.2134 × 10^−6^ m^2^ s^−1^, respectively, or 20.1% and 19.6% above PB. T10 had the lowest mean of 0.1676 × 10^−6^ m^2^ s^−1^ (−6.0%). Because diffusivity is given by *λ*/*C_v_*, the high hybrid values coincided with reduced heat storage rather than a marked increase in conductivity.

**Figure 4:**
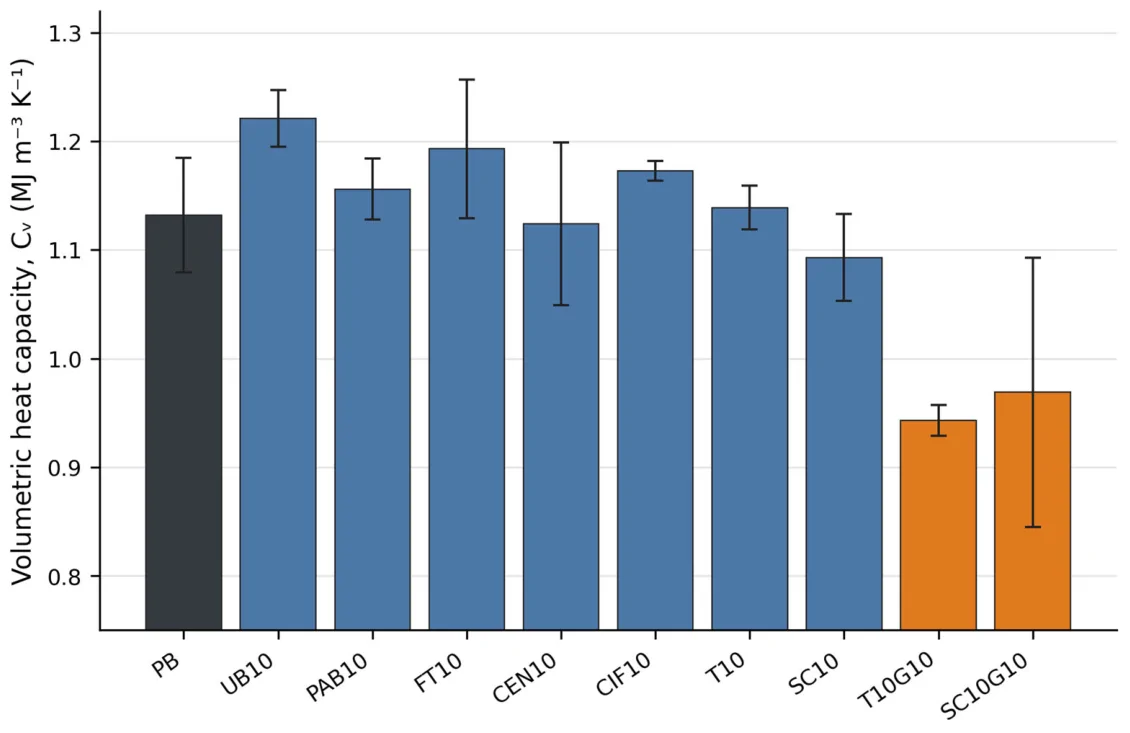
Volumetric heat capacity. Bars and capped lines denote mean ± sample SD across two specimen-pair means (n = 2).

UB10 had the largest volumetric heat capacity at 1.221 MJ m^−3^ K^−1^ (+7.8% relative to PB), followed by FT10 at 1.193 MJ m^−3^ K^−1^ (+5.3%). T10G10 and SC10G10 had the lowest means, 0.943 and 0.969 MJ m^−3^ K^−1^, corresponding to reductions of 16.7% and 14.5% (Figure 4). The same formulations consequently combined more rapid temperature propagation with less energy stored per unit volume.

**Figure 5:**
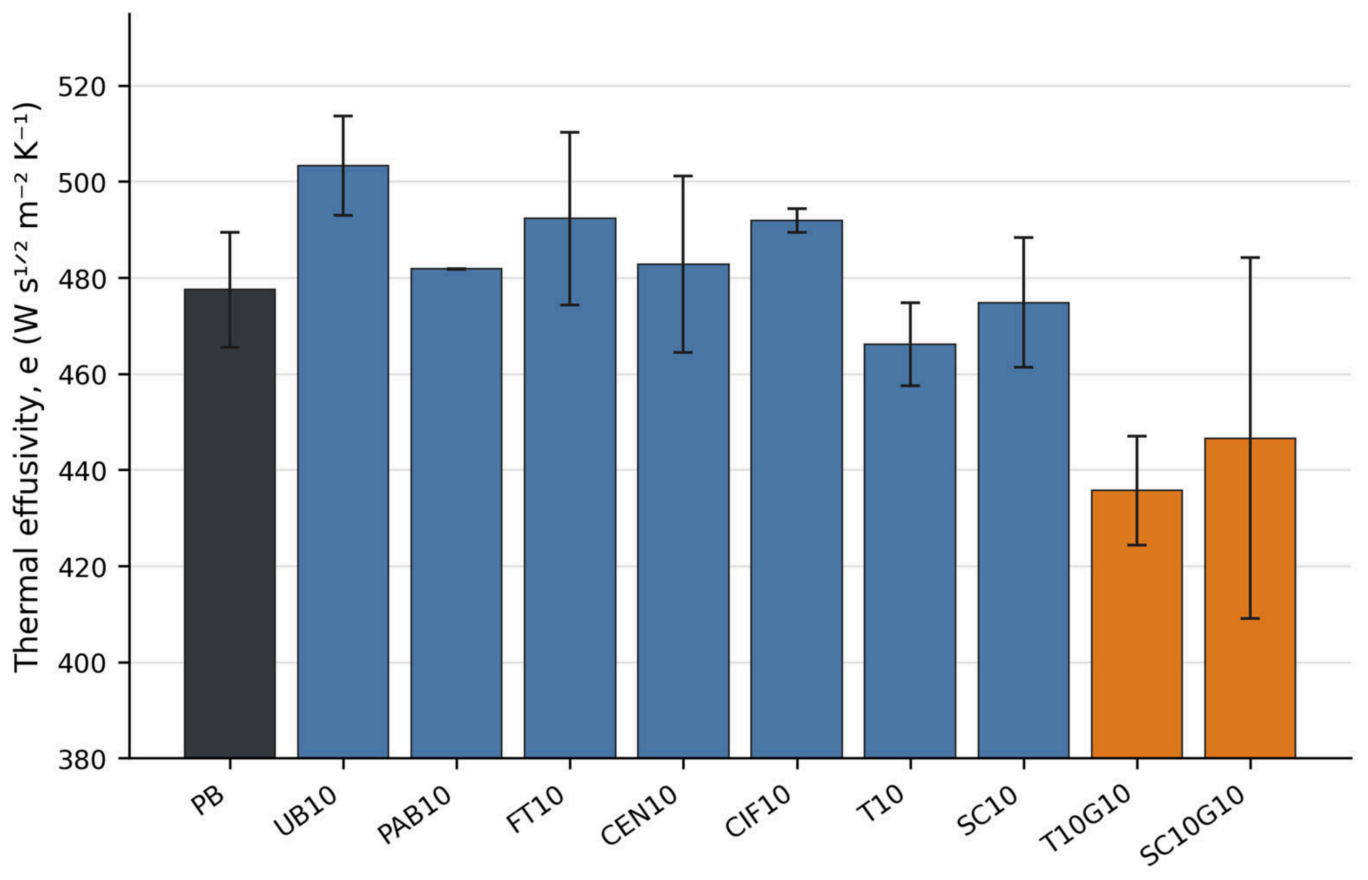
Thermal effusivity. Bars and capped lines denote mean ± sample SD across two specimen-pair means (n = 2).

Effusivity ranged from 435.7 W s^1/2^ m^−2^ K^−1^ for T10G10 to 503.3 W s^1/2^ m^−2^ K^−1^ for UB10 (Figure 5). Relative to PB, T10G10 and SC10G10 decreased by 8.7% and 6.5%, whereas UB10 increased by 5.4%. These differences describe the transient thermal contact response; they should not be read as direct measures of insulation quality.

**Figure 6:**
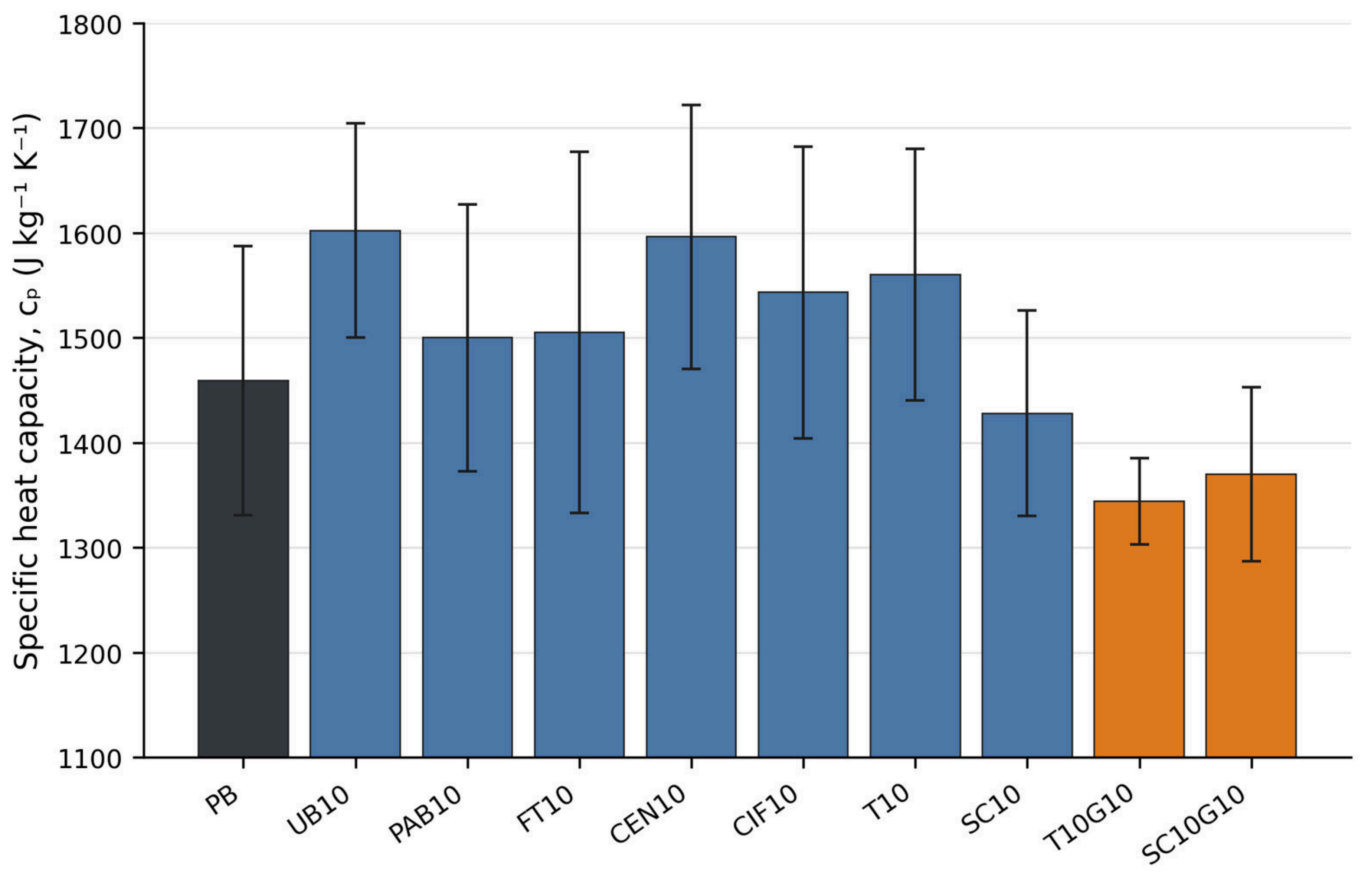
Specific heat capacity calculated for each specimen pair from *C_v_* and pair density. Bars and capped lines denote mean ± sample SD (n = 2 pairs).

Pair-level specific heat capacity ranged from 1344 to 1602 J kg^−1^ K^−1^ (Figure 6). The broad and overlapping SD intervals reflect variations in both *C_v_* and the paired density term. Reporting this uncertainty is preferable to treating the calculated *c_p_* values as exact, but the limited pair count prevents inferential comparisons.

**Figure 7:**
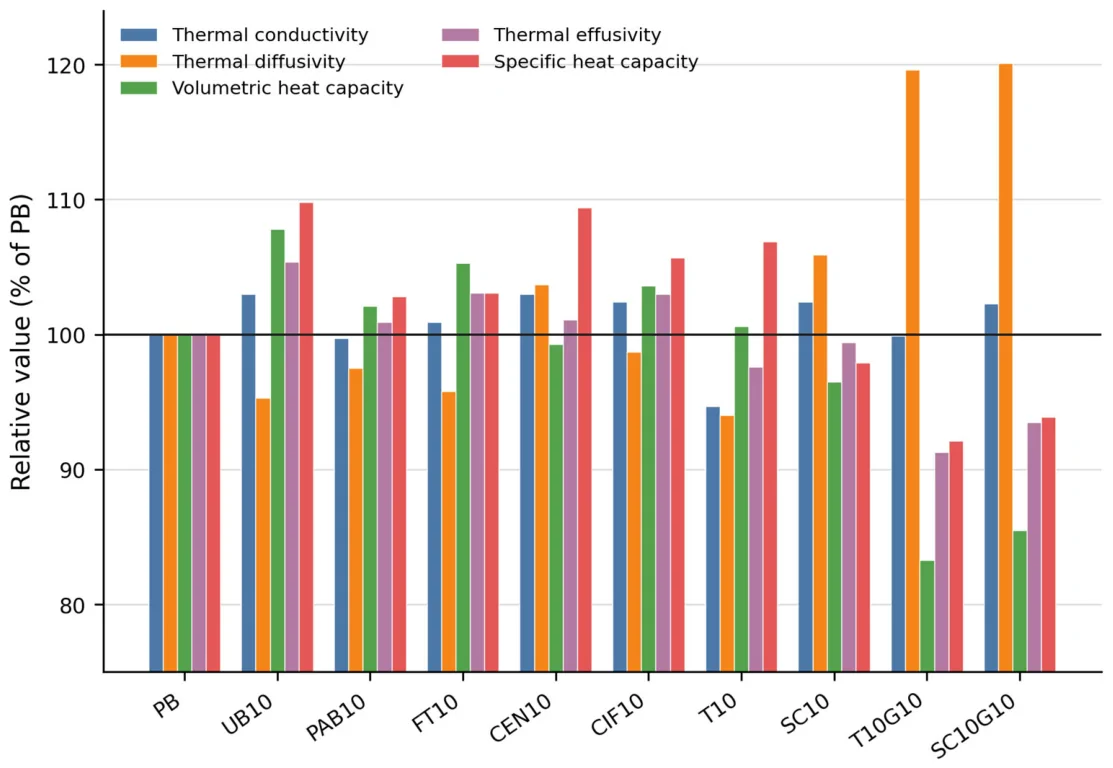
Thermophysical formulation means normalized to PB (100%). Values above or below the reference line denote increases or decreases, respectively.

The normalized comparison in Table 3 and Figure 7 makes the contrasting behavior of the hybrids clear: conductivity stayed close to PB, while diffusivity increased and both volumetric heat capacity and effusivity declined. Graphite can raise composite conductivity when its particles form connected pathways [23,24,25,26]. The present pattern offers no evidence of such a continuous network; instead, it reflects the combined composition, density, contacts, and void structure of each hybrid.

### 3.2. Normal-Incidence Sound Absorption

**Figure 8:**
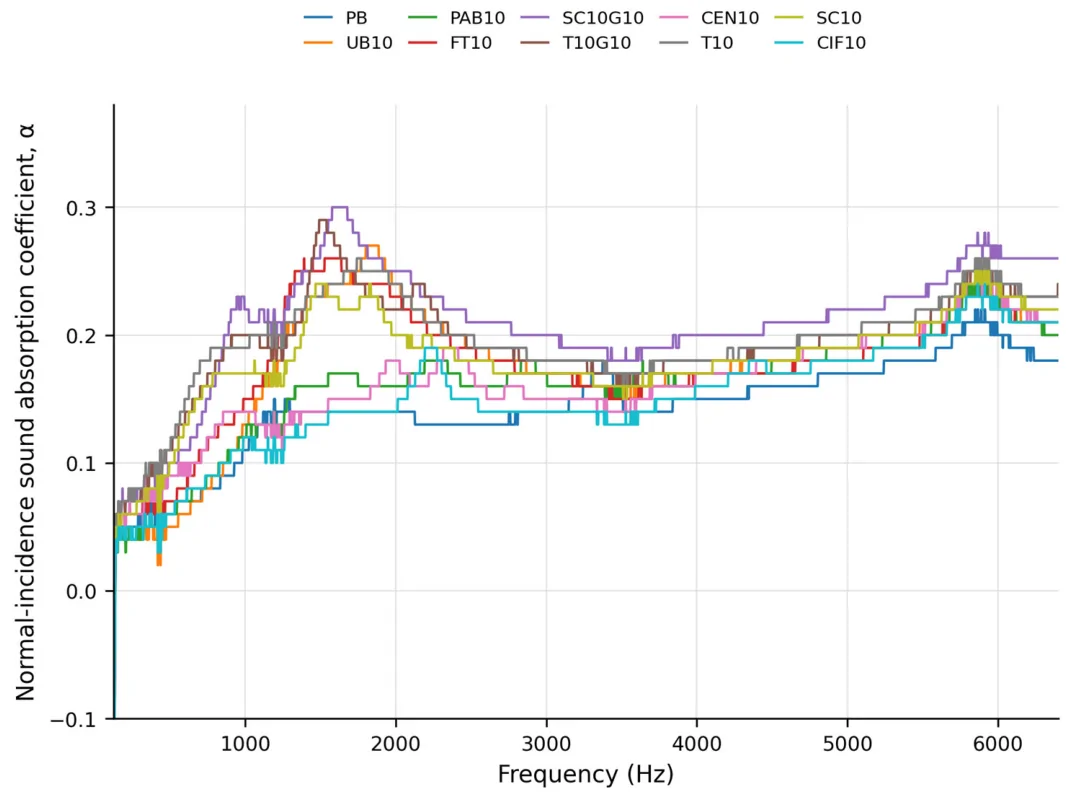
Normal-incidence sound absorption coefficients from 126 to 6400 Hz. One archived spectrum was available per formulation. Small negative values near the lower-frequency limit are retained as measurement uncertainty around zero.

Table 4 and Table 5 and Figure 8 show the single archived absorption spectrum for each formulation. Absorption was very low near the lower measurement limit and rose toward a broad maximum around 1–2 kHz before increasing again toward 6.4 kHz for several boards. Small negative values near 126–140 Hz were retained as recorded; they represent measurement scatter around an absorption coefficient of zero rather than physical sound generation.

**Figure 9:**
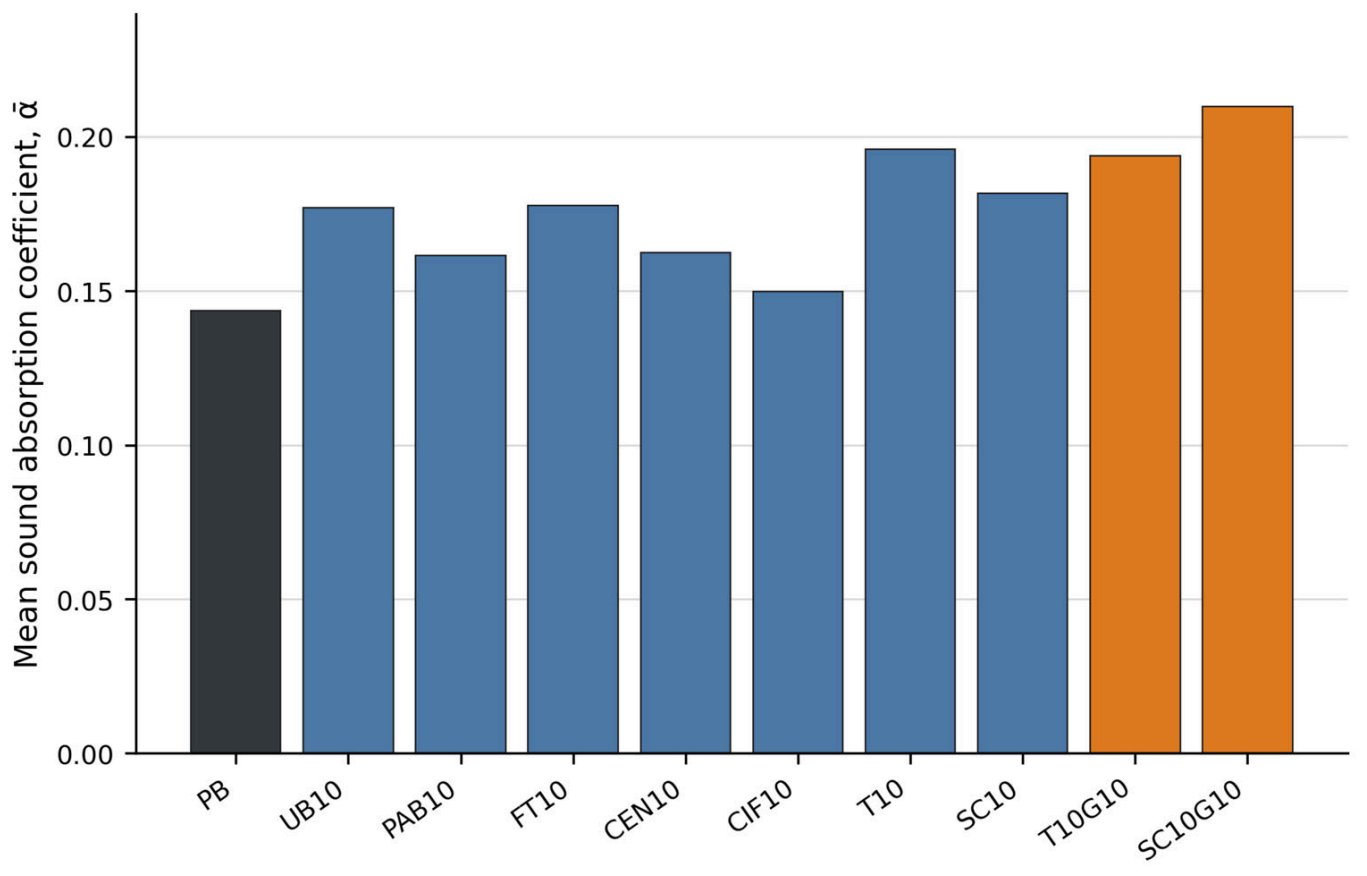
Mean normal-incidence sound absorption coefficients over 126–6400 Hz. Values are descriptive because one spectrum was available per formulation.

**Figure 10:**
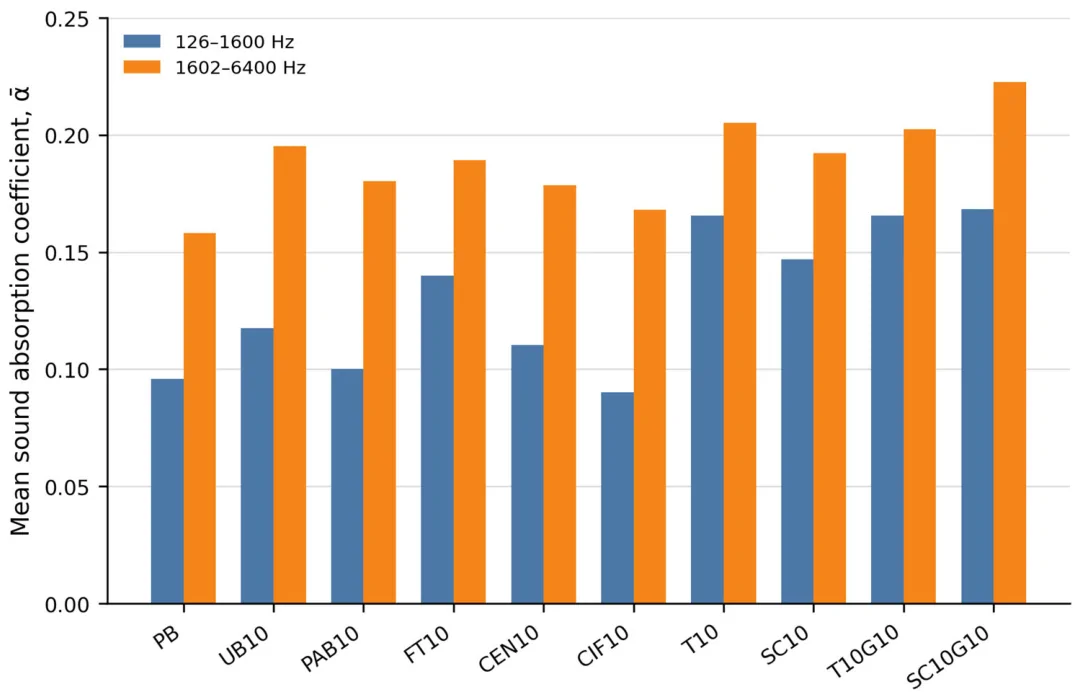
Mean normal-incidence sound absorption coefficients over the non-overlapping 126–1600 and 1602–6400 Hz bands.

Across the full range, SC10G10 had the highest mean coefficient (0.210), 46.2% above PB (0.144). T10, T10G10, and SC10 followed at 0.196, 0.194, and 0.182, respectively. The same ordering was broadly visible in the two band means (Figure 9 and Figure 10), with the largest separation from PB occurring above the lowest-frequency region.

**Figure 11:**
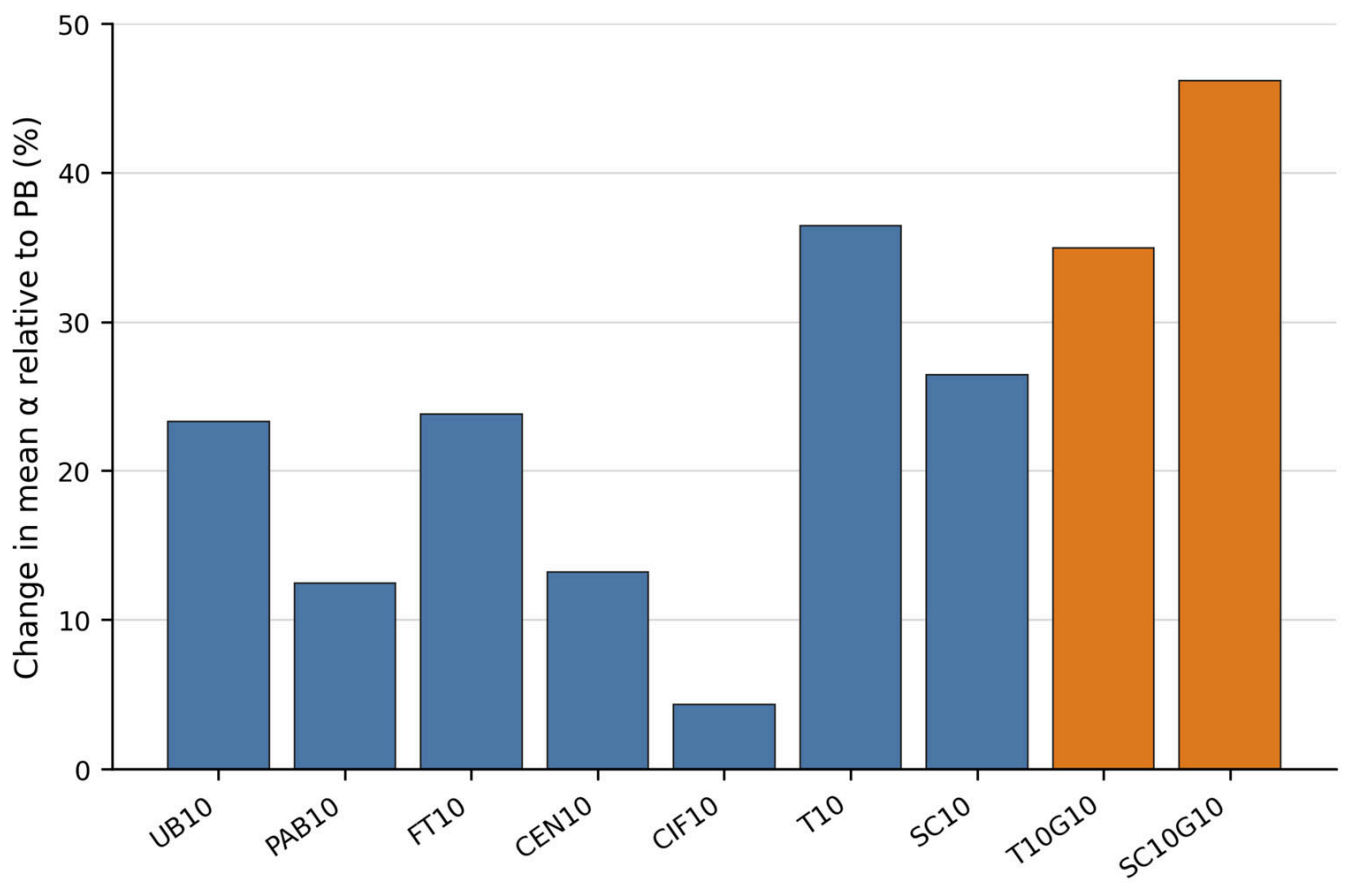
Relative changes in the mean absorption coefficients over 126–6400 Hz compared to PB.

**Figure 12:**
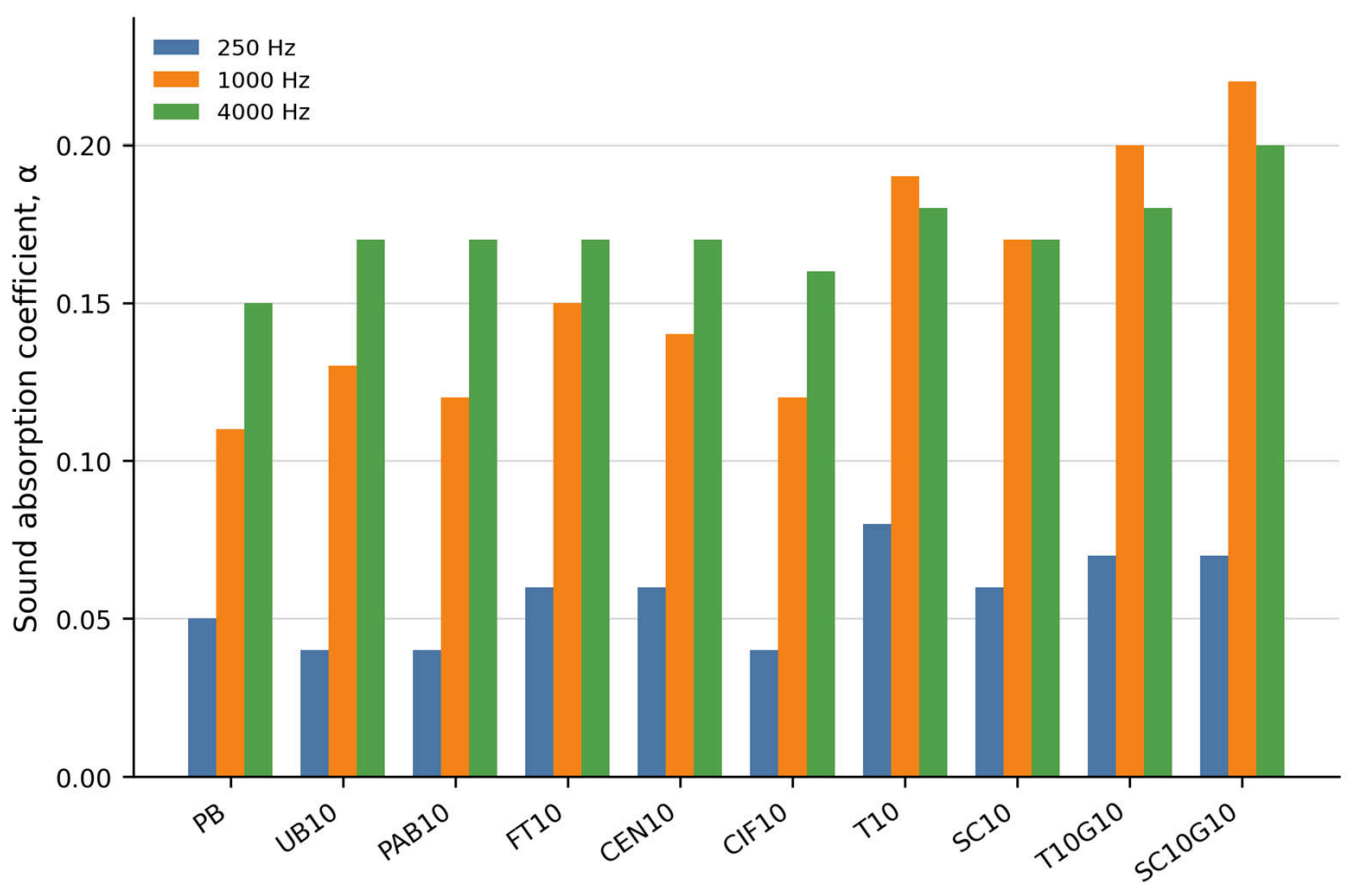
Normal-incidence sound absorption coefficients at 250, 1000, and 4000 Hz.

The full-range relative changes are plotted in Figure 11. At the selected frequencies in Figure 12, SC10G10 reached α = 0.22 at 1000 Hz and 0.20 at 4000 Hz, compared to 0.11 and 0.15 for PB. Because thickness was fixed at 18 mm and no replicate spectra were archived, these results establish formulation-specific screening differences at one thickness; they do not establish a general thickness law or statistical superiority.

### 3.3. Radiant-Heat Mass-Loss Behavior

**Figure 13:**
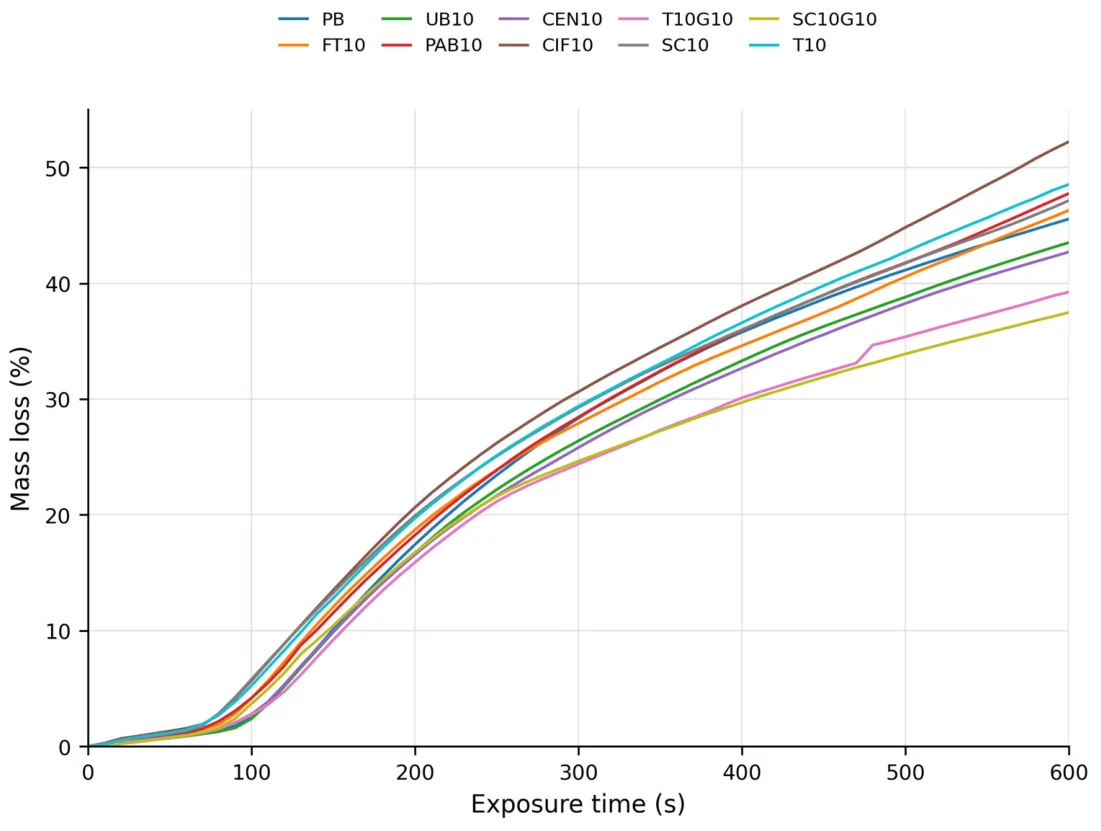
Mass loss during 600 s of exposure to 30 kW m^−2^. One specimen series was available per formulation.

All specimens showed little mass change during the initial heating interval, followed by a rapid rise and then a lower, gradually declining interval rate (Figure 13). Separation among formulations became most evident after approximately 100–150 s. At 600 s, SC10G10 and T10G10 had the lowest recorded mass losses, 37.48% and 39.24%, whereas CIF10 had the highest at 52.23% (Table 6).

**Figure 14:**
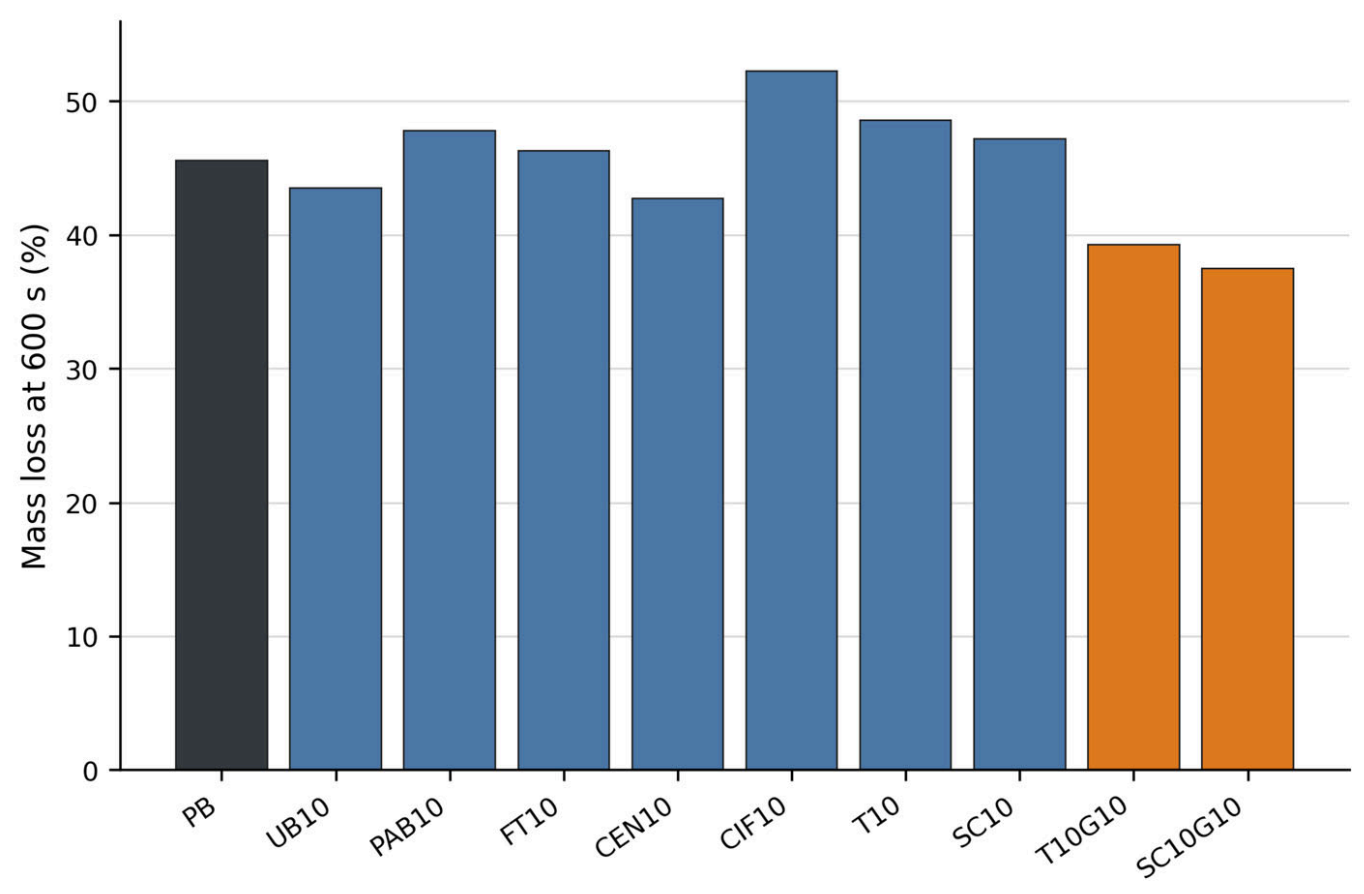
Final mass loss after 600 s of radiant-heat exposure.

**Figure 15:**
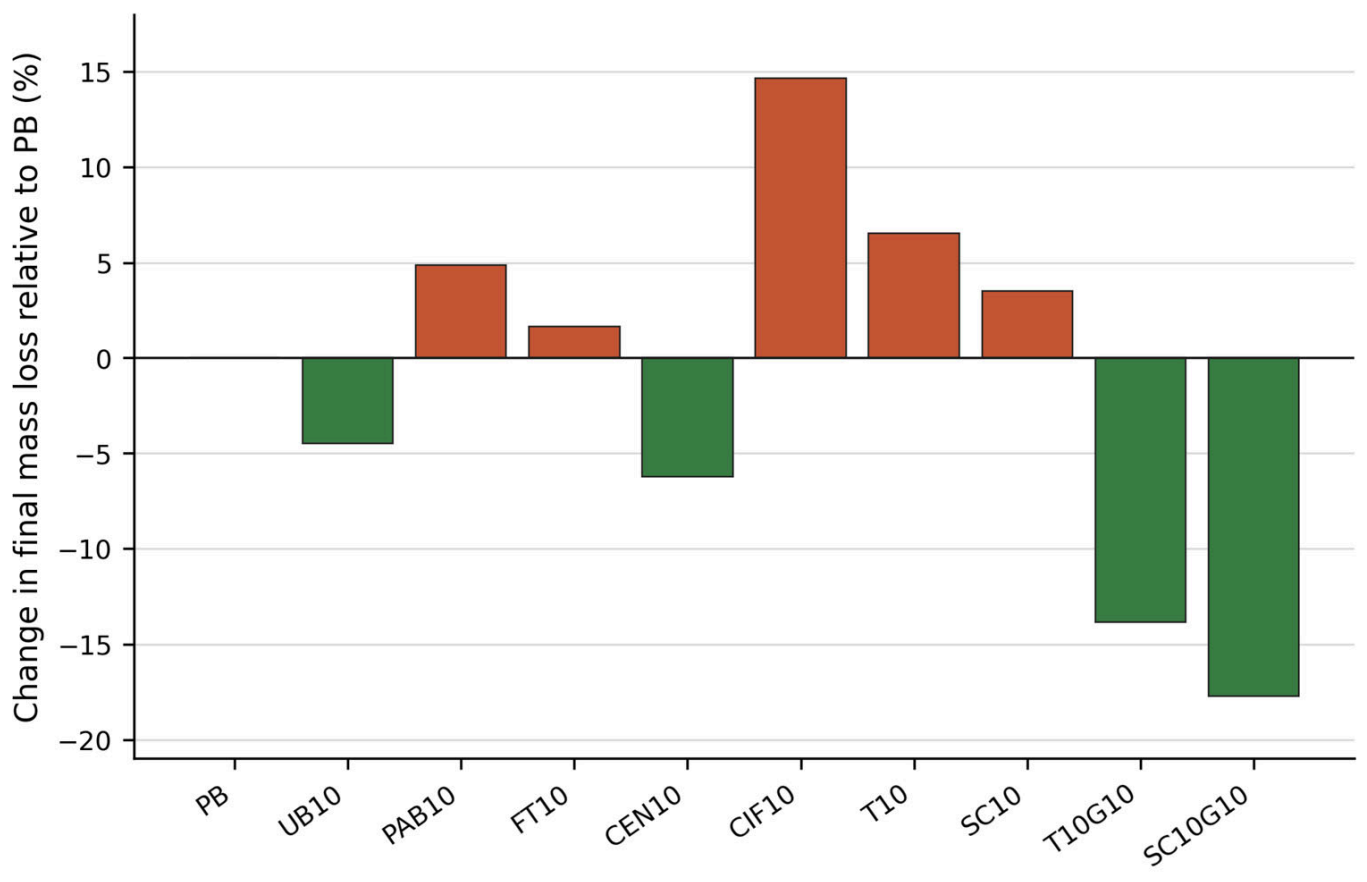
Changes in final mass loss relative to PB. Negative values denote lower recorded mass loss.

Relative to PB, final mass loss decreased by 17.72% for SC10G10, 13.85% for T10G10, 6.25% for CEN10, and 4.48% for UB10 (Figure 14 and Figure 15). PAB10, T10, and CIF10 showed increases of 4.85%, 6.55%, and 14.66%. These percentages compare one recorded specimen per formulation and should be used to prioritize replicated fire testing, not to claim fire resistance.

**Figure 16:**
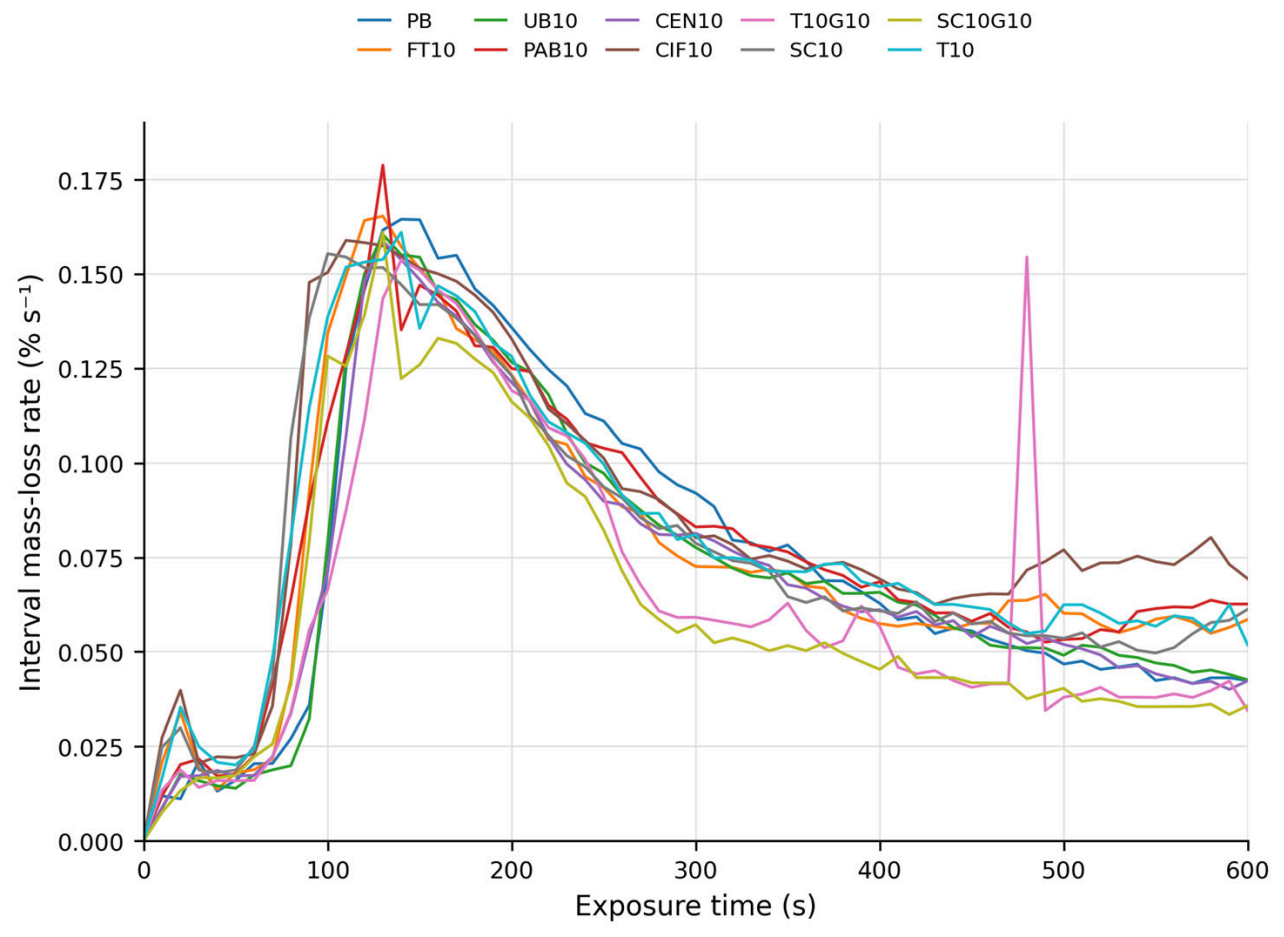
Interval mass-loss rates calculated from consecutive 10 s mass-loss measurements.

The highest interval rate was recorded for PAB10 (0.1788% s^−1^ at 130 s). Most peak rates occurred between 100 and 140 s; T10G10 showed an isolated later maximum at 480 s (Figure 16). Average rates mirrored the final mass loss because they integrate the same 600 s series: SC10G10 and T10G10 were lowest, and CIF10 was highest.

### 3.4. Macroscopic Structure and Polarizing Optical Microscopy

**Figure 17:**
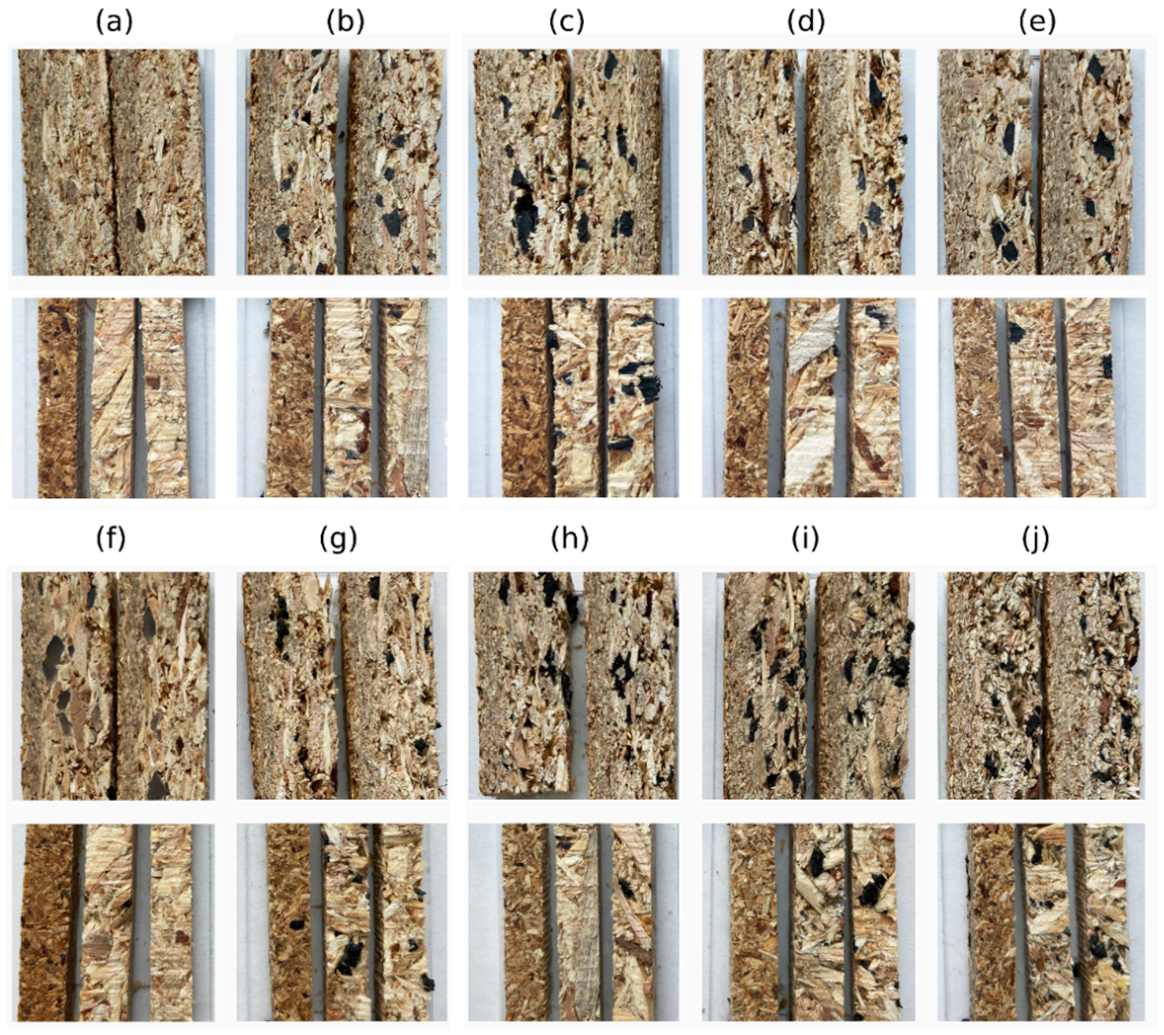
Macroscopic cross-sectional and longitudinal views: (**a**) PB, (**b**) PAB10, (**c**) UB10, (**d**) FT10, (**e**) CEN10, (**f**) CIF10, (**g**) T10, (**h**) SC10, (**i**) T10G10, and (**j**) SC10G10.

Macroscopic views confirmed the intended three-layer architecture and located most formulation-dependent heterogeneity in the core (Figure 17). Bumper- and cable-containing cores contained comparatively distinct dark granules, whereas rubber-rich and graphite formulations showed a finer, more irregular dark phase and visible spaces around some inclusions. These observations are qualitative and depend on the selected section.

**Figure 18:**
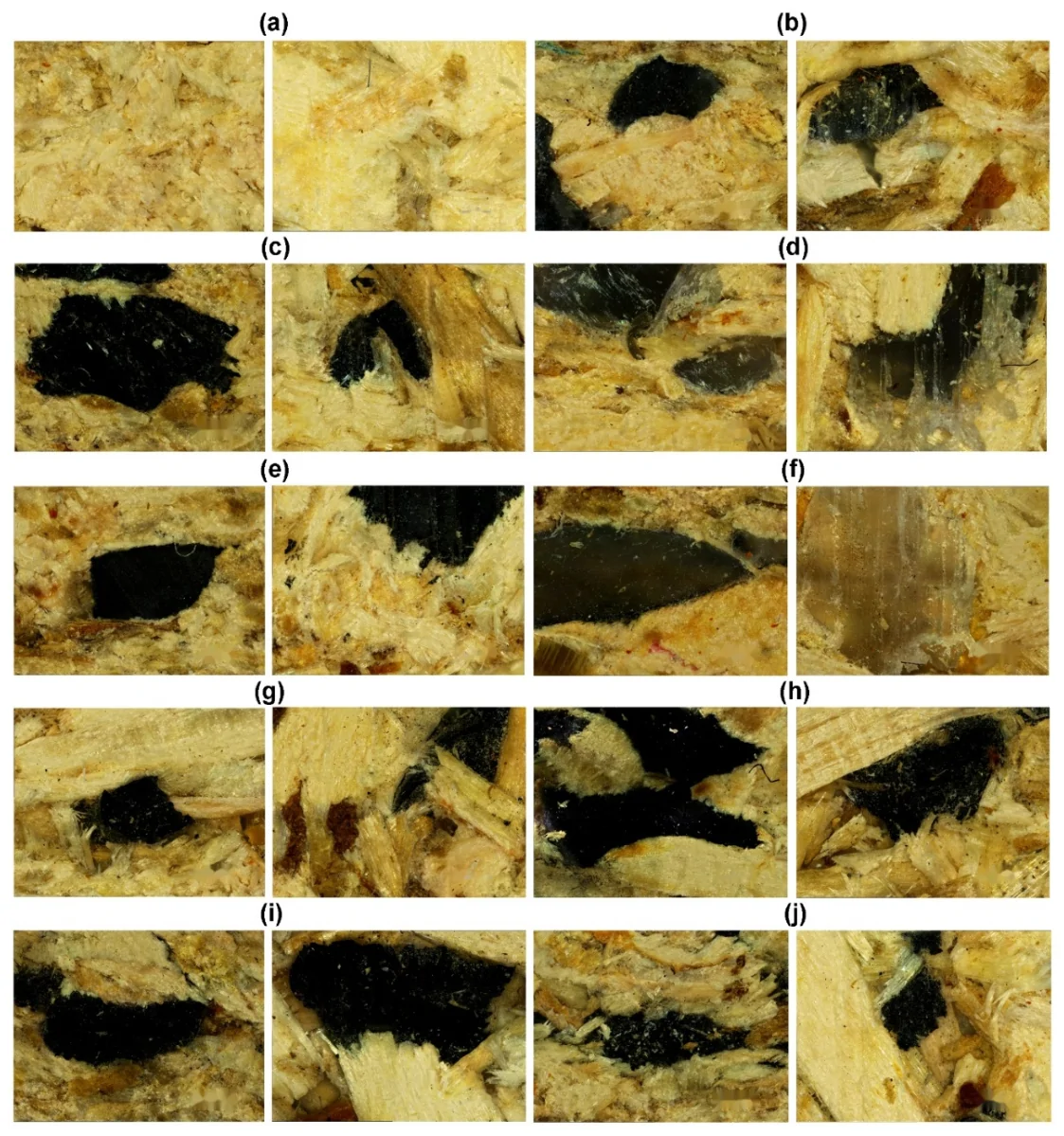
Polarizing optical micrographs at 40× (0.85 μm px^−1^): (**a**) PB, (**b**) PAB10, (**c**) UB10, (**d**) FT10, (**e**) CEN10, (**f**) CIF10, (**g**) T10, (**h**) SC10, (**i**) T10G10, and (**j**) SC10G10. Two representative fields are shown per formulation. All micrographs are shown at the same scale; scale bar = 500 μm.

At 40× magnification, the optical micrographs revealed formulation-dependent particle shape, distribution, and visible filler–wood spaces (Figure 18). PAB10 and the cable fractions generally showed sharply bounded inclusions; the tire, seal-and-carpet, and graphite systems were more heterogeneous within the observed fields. The images cannot resolve nanoscale contacts, distinguish adhesive from all constituents, or demonstrate chemical interactions.

## 4. Discussion

The most defensible thermophysical result is the contrast between relatively stable conductivity and larger shifts in the ratio between heat conduction and heat storage. Across the ten formulation means, *λ* changed by only −5.3% to +3.0% relative to PB. Czajkowski et al. reported 0.1081 W m^−1^ K^−1^ for a 637 kg m^−3^ particleboard at 8.8% moisture and 30 °C, and identified transverse and in-plane values of 0.1130 and 0.2835 W m^−1^ K^−1^ for another 18 mm, 634 kg m^−3^ three-layer board [27]. The present 0.1908–0.2075 W m^−1^ K^−1^ range falls between those directional values but is higher than the reported transverse value. This comparison is contextual rather than a validation, as density, moisture, direction, temperature, panel structure, and experimental method differ [27,28,29].

Density alone did not reproduce the ordering of mean conductivity. T10 had the lowest *λ* without having the lowest density, while UB10 and CEN10 shared the highest mean *λ* at different densities. This pattern is consistent with the known roles of pore geometry, particle contacts, resin distribution, moisture, and heat-flow direction in wood-based panels [27,28,29]. It does not prove that density was unimportant, as density SDs were large and final equilibrium moisture was unavailable, so a quantitative density–conductivity model would be underdetermined.

The hybrids are best interpreted through paired formulation contrasts. Relative to T10, T10G10 had higher α and lower *C_v_* and e, whereas relative to SC10, SC10G10 showed the same directional pattern. Conductivity changed little for SC10G10 versus SC10 and remained within the narrow study range for T10G10. Graphite-filled polymers can show large conductivity gains when dispersion and connected pathways are favorable [23,24,25]. The present optical fields show heterogeneity and visible gaps rather than evidence of a continuous network, although this hypothesis requires quantitative porosity, higher-resolution imaging, and spatial mapping.

The sound absorption screen points to rubber-rich formulations, especially SC10G10, T10, T10G10, and SC10, as candidates for replicated acoustic testing. Recycled rubber absorbers can dissipate acoustic energy through viscoelastic losses and a tortuous pore network [30,31], and recycled textiles can contribute porous absorption [32]. Those mechanisms remain plausible despite what was demonstrated here, as airflow resistivity, open porosity, dynamic stiffness, and replicate spectra were not measured. The constant 18 mm thickness permits a within-study comparison but not optimization of thickness or particle size. The independent preliminary series of Szul and Darabošová [16] likewise identified rubber-rich variants as promising candidates for further acoustic testing. This qualitative agreement across separately manufactured panel sets supports the decision to extend the work, while differences in specimens, datasets, and analytical treatment preclude pooling or direct numerical comparison.

Under the selected radiant exposure, both graphite hybrids retained more mass at 600 s than PB. Expandable graphite can form an expanded carbonaceous barrier, but the current dataset contains neither residue chemistry nor replicated heat-release measurements. The lower mass-loss curves can therefore be described as screening evidence of reduced mass degradation, not as proof of a flame-retardant mechanism or compliance with a fire classification. The distinction is especially important because final mass loss, peak interval rate, ignition, heat release, smoke, and structural fire resistance are not interchangeable endpoints [12,13,33].

Taken together, the results narrow the claims that can be made and identify the next decisive experiments. TPS measurements support pair-level descriptive differences, particularly for hybrid diffusivity and heat storage. Acoustic and radiant-heat datasets identify promising formulations but require independent specimens. Quantitative density profiling, equilibrium moisture, porosity and airflow resistivity, SEM or micro-computed tomography, chemical analysis of interfaces and residues, and mechanical and durability tests would be needed before a building-product application is proposed.

## 5. Conclusions

Replacing core-layer spruce particles with the investigated recycled fillers changed the transient response of three-layer particleboards more than their mean thermal conductivity. Pair-level conductivity means remained between 0.1908 and 0.2075 W m^−1^ K^−1^ (−5.3% to +3.0% versus PB), while T10G10 and SC10G10 increased diffusivity by 19.6% and 20.1% and reduced volumetric heat capacity by 16.7% and 14.5%, respectively. Their effusivity was 8.7% and 6.5% below PB.

At a common 18 mm thickness, SC10G10 had the highest recorded mean sound absorption over 126–6400 Hz (0.210; +46.2%). During the single-specimen radiant-heat screen, SC10G10 and T10G10 had final mass losses of 17.72% and 13.85% below PB. These acoustic and mass-loss results are useful for selecting formulations, but the absence of replicate spectra and exposed specimens precludes statistical or classification claims.

The combined evidence therefore supports formulation-dependent tuning and not a universal improvement produced by recycled waste. SC10G10 is the leading candidate for replicated acoustic and radiant-heat work, whereas T10 provides the lowest mean conductivity. Future validation should retain the specimen pair as the thermal experimental unit and add replicate acoustic and fire tests, final moisture measurements, density profiles, quantitative pore characterization, interfacial microscopy, and mechanical and durability performance.

## Figures and Tables

**Table 1:** Particleboard formulations and specimen density. Density is reported as mean ± sample SD (n = 4 specimens).

Code	Core-Layer Modification	Filler Content (wt.%)	Density (kg m^−3^)
PB	Reference particleboard; no recycled filler	0	777.4 ± 122.9
UB10	Unpainted polypropylene bumper granules	10	763.4 ± 52.9
PAB10	Painted polypropylene bumper granules	10	774.6 ± 160.9
FT10	HDPE fuel-tank granules	10	795.5 ± 106.9
CEN10	Non-flammable cable fraction (source designation)	10	704.4 ± 61.9
CIF10	Flammable cable fraction (source designation)	10	763.0 ± 96.2
T10	Tire rubber	10	732.9 ± 101.6
SC10	Seal-and-carpet residues	10	766.1 ± 73.7
T10G10	Tire rubber + expandable graphite	10 + 10	702.2 ± 30.2
SC10G10	Seal-and-carpet residues + expandable graphite	10 + 10	705.4 ± 46.1

**Table 2:** Thermophysical properties calculated from two specimen-pair means per formulation. Values are mean ± sample SD (n = 2 pairs).

Sample	*λ* (W m^−1^ K^−1^)	*α* (×10^−6^ m^2^ s^−1^)	*C_v_* (MJ m^−3^ K^−1^)	*e* (W s^0.5^ m^−2^ K^−1^)	*c_p_* (J kg^−1^ K^−1^)
PB	0.2015 ± 0.0006	0.1783 ± 0.0080	1.132 ± 0.053	477.5 ± 12.0	1459 ± 128
UB10	0.2075 ± 0.0042	0.1700 ± 0.0001	1.221 ± 0.026	503.3 ± 10.4	1602 ± 102
PAB10	0.2008 ± 0.0047	0.1739 ± 0.0081	1.156 ± 0.028	481.8 ± 0.1	1500 ± 127
FT10	0.2033 ± 0.0038	0.1708 ± 0.0060	1.193 ± 0.064	492.3 ± 18.0	1505 ± 172
CEN10	0.2075 ± 0.0018	0.1850 ± 0.0107	1.124 ± 0.075	482.8 ± 18.3	1596 ± 126
CIF10	0.2063 ± 0.0005	0.1760 ± 0.0010	1.173 ± 0.009	491.9 ± 2.5	1543 ± 139
T10	0.1908 ± 0.0037	0.1676 ± 0.0004	1.139 ± 0.020	466.1 ± 8.6	1560 ± 120
SC10	0.2063 ± 0.0043	0.1888 ± 0.0029	1.093 ± 0.040	474.8 ± 13.5	1428 ± 98
T10G10	0.2013 ± 0.0073	0.2134 ± 0.0045	0.943 ± 0.014	435.7 ± 11.3	1344 ± 41
SC10G10	0.2061 ± 0.0082	0.2141 ± 0.0191	0.969 ± 0.124	446.6 ± 37.6	1370 ± 83

**Table 3:** Thermophysical means normalized to the reference particleboard (PB = 100%).

Sample	*λ*/PB (%)	*α*/PB (%)	*C_v_*/PB (%)	*e*/PB (%)	*c_p_*/PB (%)
PB	100.0	100.0	100.0	100.0	100.0
UB10	103.0	95.3	107.8	105.4	109.8
PAB10	99.7	97.5	102.1	100.9	102.8
FT10	100.9	95.8	105.3	103.1	103.1
CEN10	103.0	103.7	99.3	101.1	109.4
CIF10	102.4	98.7	103.6	103.0	105.7
T10	94.7	94.0	100.6	97.6	106.9
SC10	102.4	105.9	96.5	99.4	97.9
T10G10	99.9	119.6	83.3	91.3	92.1
SC10G10	102.3	120.1	85.5	93.5	93.9

**Table 4:** Normal-incidence sound absorption coefficients at selected frequencies. One spectrum was available per formulation.

Sample	*α*_250_	*α*_500_	*α*_1000_	*α*_1600_	*α*_2000_	*α*_4000_	*α*_6400_
PB	0.05	0.06	0.11	0.14	0.14	0.15	0.18
UB10	0.04	0.05	0.13	0.24	0.24	0.17	0.21
PAB10	0.04	0.06	0.12	0.17	0.16	0.17	0.20
FT10	0.06	0.07	0.15	0.26	0.23	0.17	0.21
CEN10	0.06	0.09	0.14	0.15	0.18	0.17	0.21
CIF10	0.04	0.06	0.12	0.14	0.14	0.16	0.21
T10	0.08	0.11	0.19	0.24	0.24	0.18	0.23
SC10	0.06	0.10	0.17	0.23	0.20	0.17	0.22
T10G10	0.07	0.11	0.20	0.27	0.22	0.18	0.24
SC10G10	0.07	0.10	0.22	0.30	0.25	0.20	0.26

**Table 5:** Band-mean and full-range sound absorption indicators.

Sample	Mean *α*, 126–1600 Hz	Mean *α*, 1602–6400 Hz	Mean *α*, 126–6400 Hz	Change vs. PB (%)
PB	0.096	0.158	0.144	0.0
UB10	0.118	0.195	0.177	+23.3
PAB10	0.100	0.180	0.161	+12.5
FT10	0.140	0.189	0.178	+23.8
CEN10	0.110	0.179	0.162	+13.2
CIF10	0.090	0.168	0.150	+4.4
T10	0.166	0.205	0.196	+36.4
SC10	0.147	0.192	0.182	+26.5
T10G10	0.165	0.202	0.194	+35.0
SC10G10	0.168	0.223	0.210	+46.2

**Table 6:** Descriptive radiant-heat mass-loss indicators. One specimen series was available per formulation.

Sample	Mass Loss at 600 s (%)	Change vs. PB (%)	Peak Rate (% s^−1^)	Peak Time (s)	Average Rate (% s^−1^)
PB	45.55	0.00	0.1645	140	0.0759
UB10	43.51	−4.48	0.1605	130	0.0725
PAB10	47.76	+4.85	0.1788	130	0.0796
FT10	46.30	+1.64	0.1654	130	0.0772
CEN10	42.71	−6.25	0.1587	130	0.0712
CIF10	52.23	+14.66	0.1589	110	0.0871
T10	48.54	+6.55	0.1610	140	0.0809
SC10	47.15	+3.50	0.1554	100	0.0786
T10G10	39.24	−13.85	0.1545	480	0.0654
SC10G10	37.48	−17.72	0.1611	130	0.0625

## Data Availability

The original contributions presented in this study are included in the article. Further inquiries can be directed to the corresponding author.

## References

[1] United Nations Environment Programme, Global Alliance for Buildings and Construction (2025). Global Status Report for Buildings and Construction 2024/2025: Not Just Another Brick in the Wall.

[2] Ürge-Vorsatz D., Cabeza L.F., Serrano S., Barreneche C., Petrichenko K. (2015). Heating and cooling energy trends and drivers in buildings. Renew. Sustain. Energy Rev..

[3] Ozel M. (2014). Effect of insulation location on dynamic heat-transfer characteristics of building external walls and optimization of insulation thickness. Energy Build..

[4] Gustafsson S.E. (1991). Transient plane source techniques for thermal conductivity and thermal diffusivity measurements of solid materials. Rev. Sci. Instrum..

[5] He Y. (2005). Rapid thermal conductivity measurement with a Hot Disk sensor: Part 1. Theoretical considerations. Thermochim. Acta.

[6] European Commission End-of-Life Vehicles. https://environment.ec.europa.eu/topics/waste-and-recycling/end-life-vehicles_en.

[7] European Parliament and Council of the European Union (2000). Directive 2000/53/EC of 18 September 2000 on end-of-life vehicles. Off. J. Eur. Communities.

[8] Lee S.H., Lum W.C., Boon J.G., Kristak L., Antov P., Pędzik M., Rogoziński T., Taghiyari H.R., Lubis M.A.R., Fatriasari W. (2022). Particleboard from agricultural biomass and recycled wood waste: A review. J. Mater. Res. Technol..

[9] Maloney T.M. (1993). Modern Particleboard and Dry-Process Fiberboard Manufacturing.

[10] Silva Cazella P.H., de Souza M.V., Rodrigues F.R., da Silva S.A.M., Bispo R.A., De Araujo V.A., Christoforo A.L. (2024). Polyethylene terephthalate (PET) as a recycled raw material for particleboards produced from Pinus wood and biopolymer resin. J. Clean. Prod..

[11] Wronka A., Kowaluk G. (2024). Supporting circular economy principles by recycling window frames into particleboard. Materials.

[12] Lu J., Wang D., Jiang P., Zhang S., Chen Z., Bourbigot S., Fontaine G., Wei M. (2021). Design of fire-resistant, sound-absorbing, and thermal-insulated expandable polystyrene-based lightweight particleboard composites. Constr. Build. Mater..

[13] Mancel V., Čabalová I., Krilek J., Réh R., Zachar M., Jurczyková T. (2022). Fire resistance evaluation of new wooden composites containing waste rubber from automobiles. Polymers.

[14] Darabošová A., Bubeníková T., Čabalová I., Badida M., Olgun Ç., Tor Ö., Öncel M. (2025). Mechanical properties, physical properties and VOC emissions of three-layer particleboards with recycled automotive plastics in the core layer. Polymers.

[15] Čabalová I., Krilek J., Bubeníková T., Ružiak I., Nemec M., Lee S.H., Lubis M.A.R., Darabošová A., Mancel V., Kristak L. (2025). Utilization of waste tire and rubber from automobiles in the manufacturing of particleboards and evaluation of its properties. Eur. J. Wood Wood Prod..

[16] Szul T., Darabošová A., Čabalová I., Němec M. (2026). Sound absorption properties of three-layer particleboard composites modified with recycled rubber and polymer waste from the automotive sector. Adv. Sci. Technol. Res. J..

[17] (1993). Wood-Based Panels—Determination of Moisture Content.

[18] (1993). Wood-Based Panels—Determination of Density.

[19] (2022). Plastics—Determination of Thermal Conductivity and Thermal Diffusivity—Part 2: Transient Plane Heat Source (Hot Disc) Method.

[20] Bouguerra A., Aït-Mokhtar A., Amiri O., Diop M.B. (2001). Measurement of thermal conductivity, thermal diffusivity, and heat capacity of highly porous building materials using the transient plane source technique. Int. Commun. Heat Mass Transf..

[21] (2023). Acoustics—Determination of Acoustic Properties in Impedance Tubes—Part 2: Two-Microphone Technique for Normal Sound Absorption Coefficient and Normal Surface Impedance.

[22] Zachar M., Kačíková D., Majlingová A. (2021). Method for Measuring the Burning Rate and Charring Rate of Polymers with Flame Initiation and System for Performing It.

[23] Takafuji M., Kawamoto N., Hano N., Sasahara K., Nagaoka S., Ihara H. (2020). Spherical filler-promoting thermally conductive pathway in graphite-containing polymer composites for high heat radiation. J. Polym. Sci..

[24] Feng C., Ni H., Chen J., Yang W. (2016). Facile method to fabricate highly thermally conductive graphite/PP composite with network structures. ACS Appl. Mater. Interfaces.

[25] Mohammad H., Stepashkin A.A., Tcherdyntsev V.V. (2022). Effect of graphite filler type on the thermal conductivity and mechanical behavior of polysulfone-based composites. Polymers.

[26] Huang X., Jiang P., Tanaka T. (2011). A review of dielectric polymer composites with high thermal conductivity. IEEE Electr. Insul. Mag..

[27] Czajkowski Ł., Olek W., Weres J., Guzenda R. (2016). Thermal properties of wood-based panels: Thermal conductivity identification with inverse modeling. Eur. J. Wood Wood Prod..

[28] Sekino N. (2016). Density dependence in the thermal conductivity of cellulose fiber mats and wood shavings mats: Investigation of the apparent thermal conductivity of coarse pores. J. Wood Sci..

[29] Nakaya T., Yamasaki M., Fukuta S., Matsuda Y., Sasaki Y. (2019). Thermal conductivity of low-density wood composite mats. For. Prod. J..

[30] Zhou H., Li B., Huang G.S., He J. (2007). A novel composite sound absorber with recycled rubber particles. J. Sound Vib..

[31] Xu X., Wang H., Sun Y., Han J., Huang R. (2018). Sound absorbing properties of perforated composite panels of recycled rubber, fiberboard sawdust, and high-density polyethylene. J. Clean. Prod..

[32] Danihelová A., Němec M., Gergeľ T., Gejdoš M., Gordanová J., Sčensný P. (2019). Usage of recycled technical textiles as thermal insulation and an acoustic absorber. Sustainability.

[33] Vidholdová Z., Zachar M., Iždinský J., Satinová V. (2025). Evaluation of selected fire properties of recycled particleboards. Polymers.

